# Applying a gender lens to social norms, couple communication and decision making to increase modern contraceptive use in Ethiopia, a mixed methods study

**DOI:** 10.1186/s12978-022-01440-8

**Published:** 2022-06-13

**Authors:** Nandita Kapadia-Kundu, Habtamu Tamene, Minyahil Ayele, Feleke Dana, Simon Heliso, Sanjanthi Velu, Tsega Berhanu, Guda Alemayehu, Lindsey Leslie, Michelle Kaufman

**Affiliations:** 1 Johns Hopkins Bloomberg School of Public Health, Center for Communication Programs, 111 Market Place, Suite 310, Baltimore, MD 21202 USA; 2 Johns Hopkins Bloomberg School of Public Health, Center for Communication Programs, Ethiopia, Africa Avenue (Bole Road) Dembel City Center 10th Floor, P.O. Box: 26171 Code 1000, Addis Ababa, Ethiopia; 3 United States Agency for International Development (USAID/Ethiopia), 3Q57+9C7 Addis Ababa, Ethiopia

**Keywords:** Family planning, Gender equity norms, Ethiopia, Mixed methods study, Couple communication, Decision making, Social norms

## Abstract

**Background:**

Ethiopia, sub-Saharan Africa’s second most populous country has seen improvements in women’s reproductive health. The study objectives are (1) using mixed methods research, to identify determinants of contraceptive use in four regions of Ethiopia, and (2) to explore the relationship between social norms, gender equitable norms, couple communication and contraceptive use.

**Methods:**

The study includes both quantitative and qualitative methods. Researchers interviewed a total of 2770 women of reproductive age (15–49 years) in 2016 using a structured survey covering six health areas. Eligible households were identified using a multi-stage cluster-sampling technique. Using probability proportionate to size sampling, the researchers selected 10% of the proposed target woredas (24 of 240 woredas). The qualitative study included 8 rapid assessments, 16 in-depth interviews, 24 key informant interviews, and 16 focus group discussions. Qualitative data were analyzed using NVivo version 8.

**Results:**

Adjusted odds ratios were estimated for current modern family planning use among married women with logistic regression. The primary influencing factors for contraceptive use are gender equitable norms, high self-efficacy, and weekly exposure to the radio. Qualitative data indicate that the timing of contraceptive use is linked to the social norm of the desired family size of 4–5 children. Gender inequity is evident in couple communication with men controlling decision making even if women initiated conversations on family planning. A key finding based on an inductive analysis of qualitative data indicates that the micro-processes of couple communication and decision making are often dictated by male advantage. The study identified six micro-processes that lead to gender inequity which need to be further examined and researched.

**Conclusions:**

Barriers to contraceptive use include unequal couple communication and compromised decision making. Inequitable gender norms are also barriers to modern contraceptive use. The study recommends using a gender lens to study couple communication and decision making, with the goal of making both processes more equitable to accelerate the adoption of modern family planning methods in Ethiopia.

## Plain Language summary

A reproductive health study involving mixed quantitative and qualitative methods was conducted in Ethiopia in 2016–2017. The overall objectives of the study are (1) to use mixed methods research to identify determinants of contraceptive use in four regions of Ethiopia, and (2) to explore the relationship between social norms, gender equitable norms, couple communication, and contraceptive use. The survey identified gender inequitable norms as a major barrier to contraceptive use. The qualitative study further examined the gender dimension and the interlinkages between gender norms, couple communication, and decision making for contraceptive use. Data showed that gender norms related to the daily living dimension of the gender equitable men (GEM) scale are significantly associated with current contraceptive use. The qualitative component provides us with in-depth data on the daily experiences of rural Ethiopian women in the context of modern contraceptive use. We learned that social norms related to the desired number of children and the timing of first contraceptive use are interlinked. Specifically, both women and men told us that most couples do not use contraceptives until their family size is complete, after 4–5 children. Similarly, couples who opt for 2 or fewer children are termed “selfish” and “not interested in children.” Couple communication occurs within the context of decision making where men’s decisions are usually accepted, and women’s decisions are often deferred or rejected. Programs should promote respect for women’s decision-making abilities and equitable couple communication. Notably, this study finds that microprocesses of couple communication and decision making are gendered, featuring female disadvantage. Further research is required on these microprocesses of gendered couple communication.

Ethiopia has reduced its total fertility rate from 5.5 births per woman in 2000 to 4.2 births per woman in 2015 [1]. Its current use of modern contraception is 35% among married women [2]. Ethiopia’s unmet need for family planning is 22%, indicating that services have yet to reach couples in need of contraceptive products [3]. A research challenge in reproductive health is the identification of influencing factors that can accelerate the rate of adoption of modern family planning methods and reduce the unmet need for contraceptive use.

The study uses mixed methods, which include quantitative and qualitative approaches [4]. Planned in two phases, the first phase of the study included a survey of 2770 women (15–49 years). Survey data were analyzed to identify the determinants of modern contraceptive use in four regions of Ethiopia. As a next step, we designed the qualitative study to provide in-depth information related to the significant factors associated with contraceptive use, as identified through the quantitative analysis.

The overall objectives of the study are (1) to use mixed methods research to identify determinants of contraceptive use in four regions of Ethiopia, and (2) to explore the relationship between social norms, gender equitable norms, couple communication, and contraceptive use.

The value of a mixed methods approach is that contextual factors associated with modern contraceptive use, are then further explored using qualitative methods. These contextual factors include gender equitable norms, couple communication, and family dynamics.

## Background

Ethiopia, Sub-Saharan Africa’s second most populous country has seen improvements in women’s reproductive health [5, 6]. Ethiopia has made progress in reducing gender inequities through government policies and initiatives such as the Sexual and Reproductive Health Strategy (2016–2015), the National Adolescent and Youth Health Strategy (2016–2020), the Health Sector Transformation Plan (2015/2016–2019/2020) [7]. In addition, Ethiopia has taken major strides in recent years to improve girls’ education, reduce female genital mutilation, and raise the age of marriage [7].

However, much more needs to be done. Ethiopia’s continuing journey towards improvement depends on how quickly it can reduce its maternal mortality ratio (MMR), which has fallen from 752.4 maternal deaths per 100,000 live births in 2000 to 409.8 per 100,000 live births in 2015 [8]. The global sustainable development goals (SDGs) for 2030 are set such that not a single country should report an MMR greater than 140 maternal deaths per 100,000 live births [8]. A paper on global MMR trends recommends that among other interventions, countries like Ethiopia will have to focus on expanding family planning coverage and improving the quality of family planning services [9].

Exploring contextual factors associated with modern contraceptive use is essential for identifying relevant facilitators and barriers to the use of modern family planning methods. Regional variations are common given the heterogeneity of Ethiopia’s major regions. A study conducted in Bole Eco, Ethiopia identified short birth intervals (< 2 years) and having 7 plus children as the important predictors of modern contraception [10]. Both these findings point to the need for exploring social norms around the desired number of children and assessing couples’ readiness to start contraceptive use before completing their family size. For the present study, we use the principle of bounded normative influence, which explains “the tendency of social norms to influence behavior within relatively bounded, local subgroups of a social system rather than the system as a whole” [11]. The norms around contraceptive use are guided by the number of children couple’s desire. Another study calls to improve the measurement of gender constructs to be able to show the association of gender inequities and family planning [12].

### Gender and family planning

The critical role of gender in reproductive health and family planning came to the forefront with the International Conference on Population and Development (ICPD) in 1994 [13]. ICPD led to a spurt of research that established the theoretical and empirical foundations of the impact of gender equities on women’s reproductive health, intimate partner violence, and HIV [14–16]. In the context of family planning, several dimensions of gender inequalities show evidence of the association with the use of modern contraceptives including gender equitable norms, attitudes, behaviors, and modern contraceptive use [17–19]. In addition, other gender discriminatory practices such as gender-based violence and childhood, early, and forced marriage are associated with the lack of access to family planning services and low use of modern contraceptives [20–23].

While the evidence is widespread in terms of the link between gender inequity, gender norms, and various health outcomes, the number of evaluated gender transformative interventions is relatively few [24, 25]. A specific example of a successful intervention to shift gender norms in young men occurred in three low-income communities in Addis Ababa [26]. Data indicate that the “gender equity” component of interventions is crucial to shifting gender norms in a positive direction. Similarly, a community mobilization intervention with a focus on gender equity also influenced gender norms in South Africa [14].

### Couple communication

One of the objectives of this mixed methods study is to identify actionable processes at the household level that can promote equitable couple communication and decision making for contraceptive use. The literature shows a strong empirical connection between couples discussing contraceptives and the adoption of modern contraceptive methods [27, 28]. A study that asked couples separately if they discussed family planning, showed a significant effect of couple communication on family planning use, even if only one spouse reported discussing contraceptive use with their partner [29].

While gender norms exert an umbrella effect at a societal level, their influence is felt at the household level where many reproductive health related decisions occur. Women with high support of gender equitable norms are more likely to talk about family planning with their spouses compared to women with low support of gender equitable norms [30].

However, the gap in the literature lies in the unpacking of household processes such as “couple communication” and “decision making” and how programs can strengthen these processes to enable couples to adopt contraceptives. We explored how different characteristics of couple communication unfold within households. These characteristics are examined in the qualitative component of the study by using a gender equity lens. The goal is to provide a more precise understanding of how to promote equitable couple communication.

### Decision making

Among household level factors influencing contraceptive use, decision making is one of the most important. Reproductive decision making involves decisions related to when and how many children a couple wants and if they want to opt for contraceptives [31]. While early literature focused on “reproductive agency” in women, more recently agency is viewed as a limited proxy of the complex decision-making process [32].

Discordant views between spouses can lead to disputes which escalate into gender-based violence against women [33]. A quasi-experimental study with young men in Ethiopia demonstrated a shift in gender equitable norms that led to a reduction in intimate partner violence [34].

Evidence around contraceptive decision making is extensive. Many studies report that men control the decision making around contraception [35–37]. Gender restrictive norms and gender inequities are recommended as a top research priority to improve women’s health [38–42].

### Social norms

The relationship between social norms and gender norms needs to be better understood. Social norms are the wider societal level norms that influence communities. Gender norms on the other hand are a subset of norms that “define appropriate rules of interaction, relationships, and roles at all levels of the socioecological framework. They help shape power relationships, which lead to different risks and opportunities for interventions seeking to improve sexual and reproductive health [43].”

## Methods

The mixed methods study included a survey in four regions of Ethiopia (Amhara, Oromia, Tigray, and the Southern Nations, Nationalities, and People’s Region [SNNPR]), followed by an in-depth sociocultural qualitative study. Data were collected for the quantitative study from August to September 2016 in Amhara, Oromia, Tigray, and SNNPR. The qualitative data were collected in July–August 2017.

This study is part of a broader study assessing the determinants of behaviors of an integrated health promotion program. A total of 2770 women of reproductive age (15–49 years) were interviewed using a structured survey covering six health areas. Sample size was calculated to detect a 10-point difference in the proportion of modern contraceptive use from p1 = 0.40 to p2 = 0.50. Using an alpha of 0.05 and 90% power and a design factor of 1.25, the sample size per region is 672 [44]. Adding a non-response rate of 10%, the sample size per region is 676. The total sample size for four regions is 2704.

Multi-stage cluster-sampling was used to identify eligible households. Using PPS sampling, 10% of the proposed target woredas (districts) (24 of 240 woredas) were selected. Woredas are administrative units that consist of several kebeles (which are composed of approximately 500 households). Three enumeration areas were randomly selected per woreda using PPS. We conducted univariate and bivariate analyses to describe the characteristics of women in the sample and ran Pearson’s chi-square tests to assess the statistical significance between different groups such as region, age, education, and occupation. Using logistic regression analysis, we assessed the determinants of modern contraceptive use. Data analysis was conducted using SPSS version 24 (IBM, New York, NY).

### Study measures

Gender equitable norms: To measure gender equitable norms, the study used a widely tested scale, the GEM scale which was adapted for women respondents [17, 45–47]. The adapted GEM scale includes a 21-item index. The items apply to both women and men. Principal components factor analysis was used to construct the scale. The adapted scale includes four subscales related to (1) physical violence (Cronbach’s alpha 0.75), (2) sexual relationships (Cronbach’s alpha 0.72), (3) reproductive health and disease prevention (Cronbach’s alpha 0.71), and (4) domestic chores and daily life (DCDL; Cronbach’s alpha 0.83). The Cronbach’s alpha for the overall scale was 0.89. The subscales and the overall scale were divided into equal thirds and were labeled as low, medium, or high support for gender equitable norms. The GEM scale has been tested in multiple countries, including Ethiopia, Kenya, and Tanzania. Newer scales such as the cross-cultural measure towards improved reproductive and sexual health and a contraceptive autonomy scale have been recently developed [48, 49].

Self-efficacy refers to a person’s confidence in adopting or performing an action. We measured family planning self-efficacy with the following statement: “I feel confident that I can use family planning to avoid unwanted pregnancies.”

Outcome expectancies are defined “as the believed consequences of a person’s behavior. More specifically, outcome expectancies refer to the anticipation of physical, self-evaluative (or affective), and social outcomes of one’s behavior” [50]. Outcome expectancy for family planning was measured with the following statement, “The use of modern family planning methods improves the quality of my family life”.

Vulnerability Index: the level of economic vulnerability was measured using food security, shelter, education, and access to health services. The index was constructed using four items: lacked enough food to eat, lacked shelter/house to stay in, not able to afford to send children to school, and lacked the money to buy medicines/medical treatment (experienced by the participant in the past 12 months). The vulnerability index is divided into three categories, low 8–12; moderate 5–7; and high ≤ 4 with high vulnerability, indicating a greater level of poverty.

### Linking the quantitative and qualitative components of the study

In the context of this study, we used a two-step process to examine the influence of gender inequalities on reproductive health and family planning. The first step was to analyze survey data to assess if there is a significant association between gender equitable norms and family planning use. Data indicate (Table 4) that the gender equitable norms subscale related to “household chores and daily life” is significantly associated with contraceptive use. This subscale focuses on gender equitable norms within the domestic sphere. We delve deeper into this subscale during the qualitative phase to further analyze how these gender norms unfold at the family level.

The qualitative analysis enabled the in-depth exploration of couple communication and decision making from a gender perspective. Two household processes crucial for the adoption of contraceptives are spousal communication and decision making. We decided to explore couple communication and decision making through a gender lens, in the qualitative segment of the study.

### Qualitative study

Data collection for the study occurred in eight woredas (districts) in four regions of Ethiopia, Simada and Sayint in Amhara, Adwa Rural, and Tahtay Koraro in Tigray, Adaba and Jeldu in Oromia, and Dale and Damot Sore in SNNPR. Data were collected from one kebele (smallest administrative unit) per woreda.

The interviews had a duration of 40–60 min and were conducted by trained moderators. The qualitative study included 8 rapid assessments, 16 in-depth interviews (IDIs), 24 key informant interviews (KIIs), and 16 focus group discussions (FGDs). Table 1 shows the number of study participants by region and type of interview. The focus groups had an average of 9 persons which included community members (78 women and 75 men).

**Table 1:** Final sample size for the qualitative study, by region, type and number of participants

Type of participant	Number of IDIs/KIIs/FGDs/RA					Total number of participants				
	Amhara	Oromia	SNNPR	Tigray	Total	Amhara	Oromia	SNNPR	Tigray	Total
In-depth interviews (IDIs)										
Woman with child under two	4	4	4	4	16	4	4	4	4	16
Key informant interviews (KIIs)										
HEWs	2	2	2	2	8	2	2	2	2	8
HDAs	2	2	2	2	8	2	2	2	2	8
Religious leaders	2	2	2	2	8	2	2	2	2	8
Rapid assessment (RA)										
Kebele administrator	2	2	2	2	8	2	2	2	2	8
FGDs with community members										
Male	2	2	2	2	8	20	18	16	21	75
Female	2	2	2	2	8	19	19	19	21	78
Total	16	16	16	16	64	39	37	35	42	153

*HEW* Health Extension Worker, *HDA* Health Development Army

The qualitative approach draws from Grounded Theory where we assess lived experiences that include intertwined household and societal influences and provide us with data that showed the description of interconnections between influencing factors associated with modern contraceptive use [51]. Grounded theory is described as, “a set of techniques and procedures” used to assist researchers to uncover concepts and theories based on qualitative data [52]. We used a grounded theory approach to uncover local models of couple communication, social norms, and decision making and understand its associations with contraceptive use. The grounded theory approach enables an in-depth study to discover themes, patterns, and relationships. Using inductive methods, we identified emergent perspectives based on local perceptions, experiences, and practices. In addition, advanced analysis was conducted using the framework method wherein we explored a multi-level understanding of the household processes related to adoption of modern contraceptive methods through systematic data management, development of codes and identifying processes, themes and variations based on the codes [53].

The study team prepared a codebook with 13 thematic areas and 76 codes. Coding of the transcripts was done by a team of 3 researchers using NVivo version 8 (QSR International, Burlington, MA, USA).

Ethical approval was obtained for both the quantitative and qualitative studies from the Johns Hopkins School of Public Health Institutional Review Board Office and the Ethiopian Public Health Institute Scientific and Ethical Review Committee. Data collectors and supervisors received a 1-day training in ethical research procedures including informed consent, the privacy of participants, and confidentiality.

## Results: Quantitative study

We sampled women of reproductive age (15–49 years) including married, single, divorced, or widowed women in four regions of Ethiopia. Table 2 presents the socio-demographic profile of the respondents with women’s age distribution divided into one-third each, across the 3 age groupings. More than one-half of the respondents had no formal education with Tigray being the region with the highest illiteracy (64.7%). While the predominant religion is Christianity (60.8%), Islam is the next most common religion (Table 2). SNNPR and Tigray have the highest level of economic vulnerability. Over half of respondents in Oromia and Amhara are low on economic vulnerability.

**Table 2:** Sociodemographic profile of women 15–49 years from four regions of Ethiopia (N = 2770)

Demographics	Total N = 2770	Amhara N = 674	Oromia N = 688	SNNPR N = 760	Tigray N = 648
Age of participant					
15–24	933 (32.7%)	202 (28.7%)	230 (33.4%)	296 (37.1%)	205 (31.2%)
25–34	999 (36.0%)	263 (38.9%)	256 (36.0%)	250 (32.8%)	230 (35.5%)
35–49	838 (31.2%)	209 (32.3%)	202 (30.6%)	214 (30.1%)	213 (33.3%)
Education					
No formal education	1637 (57.9%)	414 (57.4%)	481 (68.9%)	321 (46.3%)	421 (64.7%)
Primary	863 (32.6%)	191 (32.1%)	168 (25.4%)	344 (41.9%)	160 (24.7%)
Secondary or higher	270 (9.5%)	69 (10.5%)	39 (5.7%)	95 (11.8%)	67 (10.6%)
Religion					
Christian (e.g., Orthodox, Protestant, Catholic)	1944 (60.8%)	439 (53.0%)	214 (40.8%)	687 (82.5%)	604 (93.2%)
Muslim	824 (39.1%)	235 (47.0%)	474 (59.2%)	71 (17.1%)	44 (6.8%)
Other (traditional)	2 (0.1%)	–	–	2 (0.4%)	–
Marital status					
Married/cohabitating	2059 (75.1%)	515 (77.0%)	566 (80.3%)	511 (68.6%)	467 (71.2%)
Divorced/widowed/single	711 (24.9%)	159 (23.0%)	122 (19.7%)	249 (31.4%)	181 (28.8%)
Vulnerability Index					
Low	1343 (50.0%)	354 (55.6%)	399 (58.9%)	321 (36.5%)	269 (41.1%)
Moderate	825 (28.9%)	178 (27.9%)	188 (27.6%)	239 (30.3%)	220 (33.8%)
High	602 (21.1%)	142 (16.5%)	101 (13.5%)	200 (33.2%)	159 (25.0%)

^a^Weighted percentages

The most used modern family planning methods in Ethiopia are injectables, followed by implants (Table 3). Other methods such as oral pills, intrauterine contraceptive device (IUCD), female sterilization, and condoms have very low usage.

**Table 3:** Current use of modern family planning methods by region

Type of family planning methods	Amhara (n = 487)		Oromia (n = 520)		SNNPR (n = 594)		Tigray (n = 458)		Total (N = 2059)	
	n	%	n	%	n	%	n	%	n	%
Injectable	186	28.1	120	18.8	157	17.5	111	17.0	574	21.5
Implants	106	18.3	36	5.5	52	7.1	60	9.4	254	10.7
Lactational amenorrhoea	11	1.8	7	1.0	40	5.0	45	6.6	103	2.8
Pills	8	1.4	7	1.2	20	2.8	7	1.1	42	1.8
IUCD	9	1.8	6	0.8	3	0.3	2	0.4	20	1.0
Current use of family planning methods	363	54.4	199	29.6	273	36.3	217	32.9	1039	41.0

The current use of modern family planning for the respondents (minus currently pregnant women) is 41% but regional variations persist (Table 3). Amhara has a significantly higher use of family planning compared to the other three regions. Tigray and SNNPR are in the 36–37% range, and Oromia has low use of modern contraceptives (Table 3). Current contraceptive use in the study matches the 2016 EDHS prevalence of modern contraceptive use [42].

### Factors associated with current modern contraceptive use

For the survey, in addition to socio-demographic factors, we explored gender equitable norms, self-efficacy, family planning knowledge, outcome expectancy, and use of contraceptive services.

About one-half the sample of women knew of 4–8 family planning methods, and 40% knew at least 1–3 family planning methods, which indicates that women have average to high levels of family planning knowledge. Overall, women in the sample reported high self-efficacy for family planning, with 46% agreeing to their ability to use family planning methods and another 37% strongly agreeing to use family planning. Outcome expectancy refers to a person’s understanding of the benefits of an action. Women have moderate (46%) to high (41%) outcome expectancy for contraceptive use.

Data on gender equitable norms indicate that almost two thirds of women (64%) supported a moderate level of gender equitable norms; only 17% of the women in the sample were high on the gender equitable norm scale. About 19% of the sample was low on the GEM scale.

### Logistic regression model

Adjusted odds ratios (AOR) were estimated using logistic regression analysis for current modern family planning use among married women in the study sample (excluding pregnant women). Details of the model are described in Table 4. The model was fitted for women’s age, education, religion, region, and the number of children under 5 years. Additionally, we explored the association of gender equitable norms and radio listening frequency with current family planning use. Lastly, determinants including knowledge of modern family planning methods, self-efficacy, and outcome expectancy were also assessed.

**Table 4:** Logistic regression model: Determinants of current use of modern contraceptives among married women 15–49 years in Amhara, Oromia, SNNPR, and Tigray (N = 1830)

Indicators	AOR	CI (95%)
Knowledge of modern family planning methods		
Knows at most 2 methods	1	
Knows 3 or more methods	2.1***	1.68–2.80
Self-efficacy to use modern family planning methods		
Low/Moderate	1	
High	2.0***	1.63–2.57
Number of children under 5 years		
0 children	1	
Has 1 child	1.7***	1.33–2.18
Has 2 or more children	1.0	0.76–1.37
Age of women		
15–24	1	
25–34	1.1	0.76–1.35
35–49	0.6**	0.43–0.82
Education		
No formal education	1	
At least primary level education	1.4**	1.15–1.88
Religion		
Muslim	1	
Christian	1.4**	1.13–1.89
Radio listening habit		
Never had a listening habit	1	
Heard at least once a week	1.4**	1.13–1.85
Gender equity norm score (DCDL)^+^		
Low	1	
Moderate	1.3*	1.06–1.77
High	1.4*	1.06–2.06
Region		
Oromia	1	
Amhara	2.9***	2.16–4.04
SNNPR	2.2***	1.59–3.13
Tigray	1.1	0.78–1.58

Self-efficacy was assessed through this statement given by those surveyed: “I feel confident that I can use family planning to avoid unwanted pregnancies.”

^+^DCDL = domestic chores and daily life; GEM subscale was measured using agreement or disagreement with five items: (1) changing diapers, giving a bath, and feeding kids is the mother's responsibility; (2) a woman’s role is taking care of her home and family; (3) the husband should decide to buy the major household items; (4) a man should have the final word about decisions in his home; (5) a woman should obey her husband in all matters

Adjusted for vulnerability index

^*^p = < 0.05, **p = < 0.01, ***p = < 0.001; Cox and Snell pseudo r2 = 0.158; Hosmer and Lemeshow test = 0.445

Knowledge of family planning methods was measured as high if respondents knew three or more modern contraceptive methods and low if respondents knew less than 3 modern contraceptive methods. Knowledge of three or more family planning methods had a significant association with current family planning use, with contraceptive use twice as likely among women with sufficient knowledge of family planning use compared to women with insufficient knowledge (Table 4). Women with high family planning self-efficacy were twice as likely to use contraceptives compared with women with low to moderate self-efficacy for modern family planning use (Table 4).

Compared to women with no children under 5, women with one child under five were more likely to be currently using family planning methods (Table 4). Current family planning use was 40% less likely among older women 35–49 years compared to women 14–24 years. Women with at least primary education were 1.5 times more likely to be currently using family planning methods compared to women with no formal education. Religion is also associated with current family planning use where Christian women were significantly more likely to be using family planning methods compared to Muslim women (Table 4). Radio listening frequency of at least once a week was associated with current family planning use. Women who listened to the radio at least once a week were 1.4 times more likely to be currently using family planning methods compared to women who did not listen to the radio (AOR 1.4, 95%; confidence interval [CI] 1.13–1.85).

Current family planning use was more likely to have been found among women with moderate (AOR 1.3, 95%; CI 1.06–1.77) to high (AOR 1.4, 95%; CI 1.06–2.06] gender equitable norms compared to women with low gender equitable norms. The gender equitable norms five-item subscale associated with contraceptive use is related to daily chores and daily life and is described in note #3 at the end of Table 4. Women from Amhara and SNNPR are more likely to use family planning compared to women in Oromia (Table 4).

The logistic regression model indicates that the factors associated with contraceptive use are knowledge of family planning, high self-efficacy, high gender equitable norms, and listening to radio once a week. The socio-demographic factors associated with family planning are age, education, religion, and region.

## Results: Qualitative study

The qualitative study was conducted after the survey. Gaps identified after the survey analysis include the lack of social variables in the quantitative study. Social norms, couple communication, and decision making were added to the qualitative study. All these influencing processes and constructs were viewed through a gender lens.

### Social norms

Social norms are important drivers of health behaviors. Often, prevalent social norms are not aligned with healthy behaviors. We explored how social norms linked to the timing of the first use of contraceptives can delay the onset of contraceptive use.

### Social norms and number of children

The overall social norm for the number of children desired in the study is skewed towards having more children. This norm is strongly prevalent across all four regions, with minor variations. When asked how many children constitute a family with “few children,” women in an FGD in Oromia replied, “Three to four children.” Data indicate that despite the introduction of family planning, the social norm related to the desired number of children remains skewed towards more children. For example, a woman in an FGD in Tahtay Koraro, Tigray, said, “Most people have 7 and 8 children,” even though some couples use contraceptives and have 3 to 4 children.

Asked if villages exist where couples have only 2 children, a health development army (HDA) member replied, “Am I going to tell you about a single household?” (Female, 56 years old, KII, HDA, Adwa Rural, Tigray). Similarly, a health extension worker (HEW) from SNNPR said only one woman in the village has 2 children: “One woman who lives here has only two children. She uses family planning Depo-Provera, and she is not interested to give birth to more children” (Female, 35 years old, KII, HEW, Damot Sore, SNNPR).

### Reasons for preference for more children

Reasons for having more children are linked to the social pressure exerted by the norm of having a big family. Study participants stated the following reasons for preference for more children:

- Respect from the community: “I come from Warza village. However difficult it is to raise more children, he (a man) will get respect from the community if he produces many offspring.” [Male, 32 years old, FGD, Damot Sore, SNNPR].
- Social pressure to have more children: “Most people have 4 or 5 children, and I have only 2 children; I am considered a selfish woman who likes her comfort. People also advise the husband to go to other wives who could have more children.” [Female, 49-years-old, FGD, Tahtay Koraro, Tigray].
- The community will speak badly about families with two children: “The community will backbite about them because they limited the number of their children to 2. They respect women who have 6 or more children.” [Female, 30, FGD, Dale, SNNPR].

### Social norms and contraceptive use

Social norm data related to contraceptive use include three dimensions: overall support for the use of modern contraceptive methods, the timing of first contraceptive use, and the acceptability of contraceptive use immediately after marriage.

Data indicate normative support for contraceptive use, specifically injectables and implants. Women seem to prefer one of these two methods and often switch between them. Female and male community members stated that the existing norm is for people to start using contraceptives after having 4 to 5 children. Data from FGDs in the four regions indicate a preference for contraceptive use after the birth of four to six children:

- “Most of the community use contraceptives after they give birth to 4 or 5 children.” (Female, 25 years old, FGD, Sayint, Amhara).
- “They use family planning after they have four or five children.” (Female, 24 years old, FGD, Jaldu, Oromia).

A few men indicated that a woman must prove her fertility before she can start using contraceptives. One of the men gave insight into the decision-making power of men vis-à-vis contraceptive use by saying, “Her husband cannot permit [it],” implying that some husbands will not allow their wives to use contraception.

### Micro-processes of couple communication

While social norms reflect the pressures that compel spouses to conform, couple communication is a process that provides opportunities for change. The gendered aspects of couple communication emerged from the qualitative analysis. They include who initiates the discussion, who controls the conversation, whose views are accepted, and finally who makes the decision. In the context of dyadic discussions, couple communication and decision making are inextricably linked. We explore the nuances of decision making in the qualitative dataset. Often the wife “defers” to the husband’s decision, but because she has participated in the decision, even if the wife prioritizes her husband’s preference, this is still measured as “joint decision making.”

Finally, in case of disagreements between the couple in rural Ethiopia, women are the ones who face severe consequences, such as being beaten by the husband. Fear of violence can lead many women to accede to the man’s viewpoint.

Men shared in an FGD in Tahtay Koraro, Tigray that women often initiate conversations related to many different topics. One participant said, “Most of the time it is women who initiate issues about their married life and plans for the future to talk and discuss with their husbands.” Another added, “For instance, a woman could tell her husband about how to sow seeds in their farmland and she could discuss with him how to use and develop seeds for next year.” A third man said, “She could talk about kinds and amounts of seeds that should have to be sowed in their farmland.” However, data indicate that even as women initiate conversations, men continue to control the decision-making process. As one woman put it, “What men say will be done immediately, while what women say will be done after a year” (Female, 38 years old, FGD, Simada, Amhara). Another participant said, “Men’s opinion always prevails. Most women here are illiterate, thus men lead their household” (Male, 60 years old, FGD, Tahtay Koraro, Tigray).

Women are often the initiators of discussions, and a husband’s acceptance of his wife’s opinion varies among households. Although some participants reported a progressive trend, with husbands paying attention to wives’ counsel, husbands still have the upper hand when making final decisions for the couple. Participants across regions reported male dominance in decision making, stating that the husband’s views and decisions are those which prevail during discussions. The normative nature of male decision making is evident in reports by both female and male participants.

Data indicates that in case of disagreements between couples, consequences can be severe for women. Decision making in the household regarding money and household purchases—such as cattle and mobile phones—and the selling of goods is a point of conflict in households where men are considered the primary decision makers. Such decisions have the potential to result in violence against women, especially when women try to intervene and question their husbands. A woman in an FGD describes the situation: “What does going against him and his command mean? For instance, if [a husband] tells [his wife] to spend a day in a specific place working [on a] specific thing, but if she talks back to him that she will not do that work… he will beat her, for she failed to keep his command and goes against his decision” (Female, 43 years old, FGD Endabagerima, Tigray).

Several female IDI participants said they usually discuss family planning with their husbands. However, as reported earlier, the result of these conversations is decision making by men. HEWs and HDAs noted inadequate dialogue exists between women and men around family planning. Some study participants did report instances where women secretly use contraceptives without discussing with or telling their husbands. Men’s desire to have more children and religious beliefs were the primary factors for the secret use of contraceptives: “[Some] never [discuss it]; the husband wants to get a child as soon as he gets married. Because of this, the wife takes family planning secretly. Even when the newborn is female, the husband needs a male child immediately, [so this is why] these women take family planning secretly” (Female, 28 years old, KII, HEW, Dale, SNNPR).

Data indicate that male dominated decision making is a common norm, as is a pattern of couple communication in rural Ethiopia. Using a gender lens facilitated the identification of low decision making by women.

## Synthesis of results

**Fig. 1:**
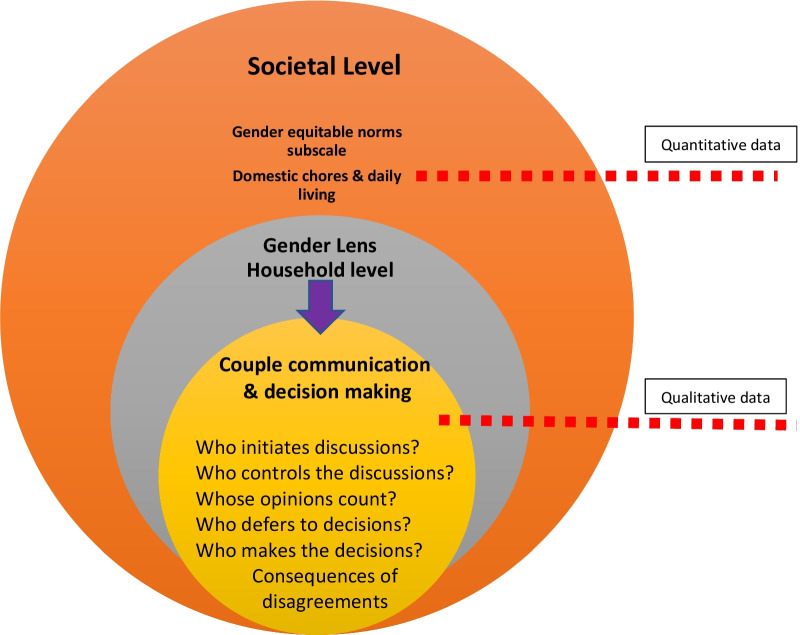
Summary of study findings. A gender lens to couple communication and decision making for increasing modern contraceptive use in Ethiopia

A summary of the results of this mixed methods study is depicted in Fig. 1. The paper provides evidence that the “domestic chores and daily living” subscale of the GEM scale is one of the significant determinants of contraceptive use. The study further explores how this subscale is operationalized at a household level. Two primary processes, couple communication and decision making were identified for in-depth study. Both these processes were studied using a gender lens (Fig. 1).

Society influences gender norms at the household level compelling families to adhere to dominant norms, specifically the timing of contraceptive use. Gender equitable norms at the household level in turn control patterns of couple communication, decision making, and access to family services.

The core learning of Fig. 1 lies in the recognition that the processes of couple communication, decision making, and access to family planning services are inherently gendered. Couple communication is a complex process that needs to be further deconstructed. The Oxford dictionary defines “process” as, “a series of things that are done in order to achieve a particular result” [54]. Micro-processes allude to the small actions that have to be undertaken to complete a process.

Using a Grounded Theory approach, we identify six micro-processes in the couple communication/decision making domain that provide a more nuanced understanding of the interpersonal discussions of couples. These micro-processes include the following:

1. Who initiates the discussion?
2. Who controls the discussion?
3. Whose opinions count?
4. Who defers to another’s decisions?
5. Who makes decisions?
6. What are the consequences if the couple disagrees?

In the context of the present study, women often initiate discussions, but men control them; usually male opinions count, and men make decisions, with women deferring to those decisions. In case of differences between the couple, the wife can face physical violence if she goes against her spouse’s will. The promotion of gender equity in couple communication and decision making is critical if women’s voices are to be heard in the context of family planning. Gender hierarchies often dictate the flow of conversation between spouses and prevent women from expressing their needs, opinions, and preferences. Similarly, data indicate that men usually retain the power of decisions. In such a scenario, examining couple communication and decision making as constructs devoid of gender implications is to miss out on ground-level realities. Finally, the combined impact of gender restrictive norms with socio-demographic and structural factors, are what influences access to the health system and family planning services.

## Discussion

This mixed methods study takes a multipronged approach to identify and contextualize determinants related to modern contraception use in four regions of Ethiopia. The study identifies the influencing factors that can determine a rapid rate of adoption of modern contraception, as Ethiopia moves towards meeting its SDGs.

The overarching construct related to contraceptive use are gender equitable norms. They represent the societal dimensions that drive the intermediary processes of couple communication and decision making. In this study, we learned how to interconnect individual, household, and social factors to obtain a nuanced understanding of the pathway to modern contraceptive use in Ethiopia. For example, the identification of gender equitable norms in the survey led to further exploration of processes of gendered couple communication and decision making in the qualitative study.

The new dimension added by this study is highlighting the need for a gender lens for viewing couple communication and decision making. We learn that unless we examine couple communication and decision making from a gender equitable perspective, equalizing the inherent gender imbalance in these constructs will be difficult. Six gendered characteristics of couple communication and decision making emerged from this study (refer section on synthesis of results). In all six characteristics, the power dynamics between the couple are evident. We need further research on the power dynamics within the two household processes studied in this paper.

Increasingly, there seems to be a global shift in the recognition of gender norms influencing family planning and other health outcomes [17]. Our study findings are aligned with global trends that focus on gender norms [40–42]. The study has demonstrated that by using a robust tool (the GEM scale, adapted for women), we add to the existing literature that has already shown an empirical linkage between gender equitable norms and modern contraceptive use [25, 26]. To further unpack household processes, our study suggests a gender dimension should be added to each intermediary construct in the behavioral pathway.

The relevant social norms for contraceptive use include norms related to the timing of contraceptive use and desired family size. The social norm related to the timing of contraceptive use needs to be further studied as there is scarce literature from Africa on this issue. Data from France on the timing of contraceptive use indicate that early initiators of contraceptive use have better reproductive outcomes [53]. Overall, there’s need for the use of modern family planning methods in the four regions. However, for this to happen, the required normative shift will involve couples who start using contraceptives much earlier and space out the births of their first four children.

Steps towards operationalizing these findings into strategic behavior change programs include conducting more nuanced research on the gender power imbalance in couple communication and decision making. Such research will identify gendered patterns of couple communication and decision making in different settings. Based on contextual findings, interventions can be designed specifically to address the gaps in equitable couple communication and decision making related to contraceptive use. For example, in the present study, we know that decision making is squarely in the hands of men even though women initiate discussions. In such a context, promoting “respect” for women’s opinions and decision-making will lead to women’s voices being heard in her own home as well as her decisions being accepted. While superficially, couples may seem to be “communicating,” an in-depth analysis indicates that a basic gender inequity dominates these interactions. To rectify the gender imbalance in couple communication and decision making, men and young boys need to be an integral part of gender-centered initiatives to promote gender equitable norms [26].

Strategic behavioral approaches can accelerate the rate of adoption of family planning methods. For example, the data identified that women with 2 children should be the audience of health promotion programs, as they have lower contraceptive use than women with 3 or more children.

### Study limitations

The study has limitations. Since the research is a part of an integrated health study, we could not include more details on reproductive health. The study is based on cross-sectional research and can only suggest empirical associations from survey data which then need further study. For the six micro-processes of couple communication, additional research will be required to see if these micro-processes will be empirically supported in different contexts. The study is exploratory in nature and does not claim any causality. It does not address supply side factors. The study was conducted from 2016–2017, so this is the time frame that should be considered when interpreting the findings. When researchers ask about social norms, a social desirability bias may be likely. Another bias is the omitted variable bias. However, the mixed method study illustrates how to combine research methods to achieve the goal of identification of contextual determinants of modern contraceptive use.

## Conclusions

The pathway to the adoption of modern contraceptive use is determined by overriding gender factors that influence the reproductive health behavior of couples. Restrictive gender norms emerged as the underlying connecting theme between intermediary influencing factors of modern contraceptive use. With SDGs being a major challenge for Ethiopia in the coming years, efforts to enable equitable couple communication and decision making are needed to increase the uptake of family planning services and the use of modern contraceptives [55].

## Data Availability

The datasets generated and/or analyzed during the current study are not publicly available, as the study protocol states, “Only study investigators will have access to the data.” They are available on a case-by-case basis upon reasonable request directed to the corresponding author.
